# A rare missense variant in *APC* interrupts splicing and causes AFAP in two Danish families

**DOI:** 10.1186/s13053-020-00140-3

**Published:** 2020-04-07

**Authors:** Malene Djursby, Karin Wadt, Jane Hübertz Frederiksen, Majbritt Busk Madsen, Lukas Adrian Berchtold, Jane Preuss Hasselby, Gro Linno Willemoe, Thomas v. O. Hansen, Anne-Marie Gerdes

**Affiliations:** 1 Department of Clinical Genetics, Rigshospitalet, Copenhagen University Hospital, Copenhagen, Denmark; 2 The Danish HNPCC Register, Clinical Research Centre, Copenhagen University Hospital, Hvidovre, Denmark; 3 Department of Genomic Medicine, Rigshospitalet, Copenhagen University Hospital, Copenhagen, Denmark; 4 Department of Pathology, Rigshospitalet, Copenhagen University Hospital, Copenhagen, Denmark

**Keywords:** *APC*, Missense variant, Splicing, Polyposis, Adenocarcinoma, Colorectal cancer

## Abstract

**Background:**

We report the first case of a missense variant in the *APC* gene that interrupts splicing by creating a new cryptic acceptor site. The variant, c.289G>A, p.(Gly97Arg), is located in exon 3, and qualitative and semi-quantitative RNA splicing analysis reveal that the variant results in skipping of the last 70 nucleotides of the exon, which leads to the introduction of a frameshift and a premature stop codon.

**Case presentation:**

The variant was detected in two, apparently unrelated, Danish families with an accumulation of colorectal cancers, colonic adenomas and other cancers. The families both have an attenuated familial adenomatous polyposis phenotype, which is consistent with the association of pathogenic variants in the 5′ end of the gene.

One variant-carrier also had Caroli Disease and a Caroli Disease associated hepatic mucinous cystadenocarcinoma. This is the first description of a person with both Caroli Disease and a pathogenic *APC* variant, and although the *APC* variant is not known to be connected to the development of the hepatic malformations in Caroli Disease, it remains unclear whether the variant could have contributed to the carcinogenesis of the liver tumour.

**Conclusions:**

Based on functional and co-segregation data we classify the *APC* c.289G>A, p.(Gly97Arg) variant as pathogenic (class 5). Our findings emphasize the importance of a functional evaluation of missense variants although located far from the exon-intron boundaries.

## Background

Adenomatous Polyposis Coli (*APC)* related disorders include familial adenomatous polyposis (FAP), attenuated FAP (AFAP) and Gastric Adenocarcinoma and Proximal Polyposis of the Stomach (GAPPS). Whereas FAP and AFAP can result from intragenic mutations, partial- or whole-gene deletions, GAPPS is only known to be caused by variants in promoter 1B of *APC*. The conditions are inherited in an autosomal dominant manner with varying degrees of penetrance from almost 100% in classic FAP to 70% at age 80 in AFAP [1–3].

Especially FAP, and to a lesser degree AFAP, are in addition to colonic adenomatous polyposis and increased risk of colorectal cancer (CRC) also characterized by an increased risk of extracolonic cancers, especially duodenal, ampullary and thyroid carcinomas, as well as non-malignant conditions and benign neoplasms. The symptoms from FAP to AFAP form a continuum, but FAP is often defined as the presence of > 100 colonic adenomas, early development of colonic adenomas (usually during the second decade) and early onset of CRC with an average age of 40 years at the time of diagnosis. AFAP, on the other hand, is typically characterized by a lower burden of colonic adenomas (most often < 100) with a tendency to right-sided localization and rectal sparing, and higher age at diagnosis [4]. AFAP has in several studies been associated with truncating pathogenic variants in the 5′ and 3′ end of the *APC* gene as well as exon 9, although the codons involved have varied slightly. A clear genotype-phenotype correlation is still controversial [5–7].

Here, we report two families with an extremely rare, pathogenic *APC* missense variant and AFAP phenotype.

## Case presentation

**Fig. 1:**
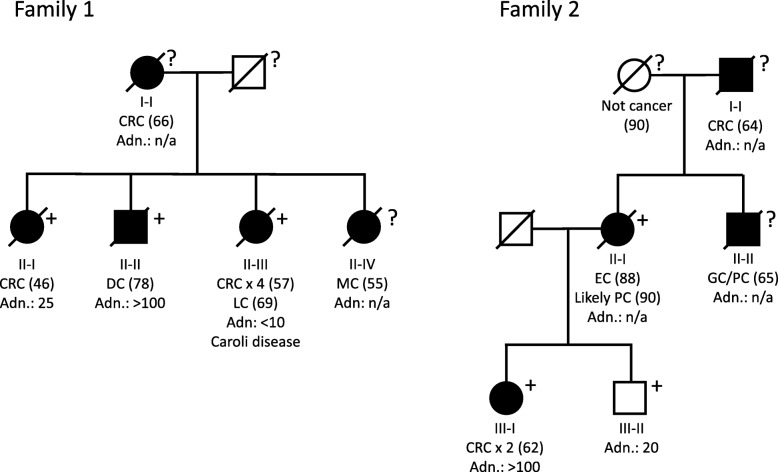
Pedigree of family 1 and 2, and segregation analyses of the *APC* variant c.289G>A, p.(Gly97Arg). *Abbreviations: CRC: Colorectal cancer, adn: Colonic adenomas, DC: Duodenal cancer, LC: Hepatic cancer, MC: Breast cancer, EC: Endometrial cancer, PC: Pancreatic cancer, GC: Gastric cancer, n/a: Not available. Numbers in () refer to age at diagnosis (or death, if the person did not develop cancer). +: APC variant c.289G>A carrier, ?: APC variant c.289G>A carrier status unknown*

The first family (family 1) was included in a research project aimed at identifying new CRC genes, or genes not previously known to be involved in CRC, in families with an accumulation of CRC, and with no identified pathogenic variant in the DNA mismatch repair (MMR; *MLH1*, *MSH2*, *MSH6* and *PMS2*) genes. The family was identified through the Danish HNPCC Register, Clinical Research Centre, Copenhagen University Hospital, Hvidovre. Two siblings (individual II-I and II-II); one with an adenocarcinoma in the transverse colon at age 46 and one with an adenocarcinoma in the duodenum at age 78, were included. They also both had colonic adenomatous polyposis. Another sibling (II-III) was later included in order to perform segregation analysis in the family. She was affected by four primary CRCs and a hepatic mucinous cystadenocarcinoma (Fig. 1; Table 1) and had also been diagnosed with the rare condition Caroli disease (CD). According to the family records, many relatives have suffered from gallstones and have had the gallbladder removed at an early age – as early as the beginning of the third decade - however, gallbladder disease do not segregate with CRC in the family. *MLH1*, *MSH2*, *MSH6* and *APC* had previously, in 1999, been tested in the family, but for unknown reasons the *APC* variant was not detected or interpreted as pathogenic.

**Table 1:** Family characteristics

Individual ID	Gender	Age: Cancer localization (pathology)	Colonic adenomas	Mutation status (***APC***)	Comments
**Family 1**					
**I-I**	Female	66: Colon, hepatic flexure (AC)	n/a	n/a	Cholecystitis chronica without specification.
**II-I**	Female	46: Transverse colon (AC)	> 25	c.289G>A	46: Colectomy
**II-II**	Male	78: Duodenum (AC)	> 100	c.289G>A	64: Prophylactic subtotal colectomy
**II-III**	Female	57: Descending colon (AC)	< 10	c.289G>A	57: Subtotal colectomy.
		57: Rectum (AC)			
		57: Transverse colon (AC)			
		69: Intrahepatic tumor (MCAC)			Caroli disease.
**II-VI**	Female	55: Breast (IDC)	n/a	n/a	21: Cholecystectomy.
**Family 2**					
**I-I**	Male	64: Caecum (AC)	n/a	n/a	
**II-I**	Female	88: Uterus (EA)	> 30	c.289G>A	*) Suspected based on a CT-scan.
		90: Likely pancreatic cancer*			
**II-II**	Male	65: Liver metastases with primary tumor in the gastrointestinal tract, most likely pancreatic or gastric cancer (pathology n/a)	n/a	n/a	
**III-I**	Female	62: Sigmoid colon (AC)	Multiple, likely > 100	c.289G>A	62: Total colectomy.
		62: Sigmoid colon (AC)			
**III-II**	Male	–	< 20	c.289G>A	64: No cancer diagnosis.

*Abbreviations*: *AC* adenocarcinoma, *MCAC* mucinous cystadenocarcinoma, *IDC* invasive ductal carcinoma, *EEA* endometrioid adenocarcinoma, *n/a* not available

The second family (family 2) was referred to Department of Clinical Genetics due to an accumulation of gastrointestinal cancers and adenomas. The proband (individual III-I) had a total colectomy at age 62 due to multiple (> 100) colonic adenomas, and the pathological evaluation revealed adenocarcinoma in two of the adenomas. A colonoscopy on her 64-year-old brother (III-II) revealed that he had approximately 20 adenomas. The family history included at least three additional cases of gastrointestinal cancers (Fig. 1; Table 1).

All living individuals received written and oral information as well as genetic counselling, and a written informed consent was obtained. As for the research project, ethical approval was obtained from the Danish Committee on Health Research Ethics (reference: H-4-2014-050).

In individual II-I and II-II, family 1, we analysed a research gene panel consisting of 34 defined or candidate genes for colorectal cancer. In individual III-I, family 2, we analysed a clinical CRC gene panel consisting of 17 CRC/polyposis predisposing genes. Both panels included *APC* (supplementary table S1 provides a complete list of the included genes). The analyses were next-generation sequencing based (Illumina, San Diego, California, USA) and performed on germline DNA obtained from peripheral blood.

In all three individuals we identified the *APC* variant c.289G>A, p.(Gly97Arg). Subsequent segregation analyses showed that a third sibling (II-III) in family 1 also carried the variant, as well as two individuals in family 2 (II-I and III-II); pedigrees can be seen in Fig. 1. Several healthy individuals from family 1 have also been tested, none of whom had inherited the variant; they were all following a surveillance program with regular colonoscopies without evidence of polyposis.

The *APC* c.289G>A variant has to the best of our knowledge only been published once in a Chinese patient with mild FAP [8], but has not been reported in any population allele frequency database (dbSNP, ExAC or gnomAD) including in 2000 exomes from Danish patients with diabetes [9]. In silico splice prediction software (SpliceSiteFinder-like, MaxEntScan and NNSPLICE; all integrated in the Alamut visual software (v.2.11)) all predicted that a strong cryptic acceptor splice site was introduced 70 base pairs into exon 3, which could lead to aberrant splicing and a lack of 70 nucleotides in the mRNA and probably nonsense mediated mRNA decay.

To confirm this hypothesis, we analysed RNA isolated from whole blood samples from individual III-I (family 2) by qualitative RT-PCR and semi-quantitative capillary electrophoresis (CE) using primers located in exon 1 and exon 5, respectively (primer sequences are available upon request).

**Fig. 2:**
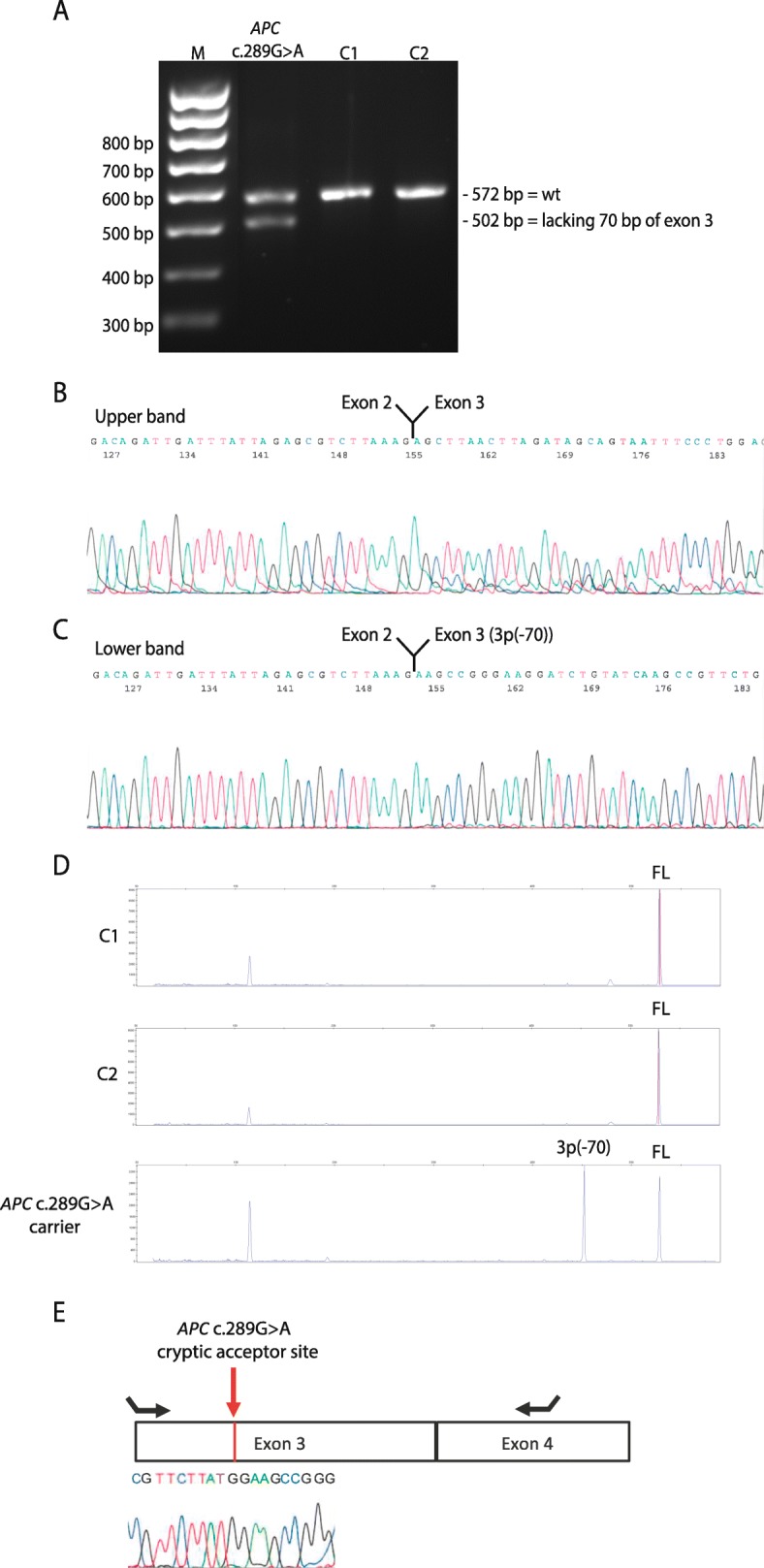
RNA and allele-specific analyses of the *APC* c.289G>A variant. **a** Gel electrophoresis analysis of RT-PCR products from an *APC* c.289G>A carrier and two controls (C1 and C2). **b** Sanger sequencing of the upper RT-PCR product. **c** Sanger sequencing of lower RT-PCR product. **d** Capillary electrophoresis analysis of the RT-PCR products from an *APC* c.289G>A carrier and two controls (C1 and C2). FL: Reference full-length transcript, 3p(− 70): Transcript lacking 70 bp of exon 3. **e** Sanger sequence of an RT-PCR product using primers designed to target the skipped region in exon 3 and to amplify the heterozygote c.289G>A variant in the carrier. The Sanger sequence show a monoallelic contribution of the wt allele

The RT-PCR analysis detected two major transcripts in the *APC* c.289G>A carrier, while only one transcript was identified in the two control samples (Fig. 2a). Sanger sequencing revealed that the upper transcript corresponded to the reference full-length (572 bp), while the lower band represented a transcript lacking 70 bp of exon 3 (502 bp (3p-70), Fig. 2b, c). Semi-quantitative CE supports the RT-PCR data and showed the control samples only expressed the full-length transcript, while the carrier expressed the full-length and a transcript corresponding to exon 3 lacking 70 nucleotides (3p(− 70)). The data moreover indicated that the 3p(− 70) transcript contributed to ~ 50% of the total transcript expression in the carrier (Fig. 2d). To further determine the contribution of the c.289G>A variant allele to *APC* reference transcript expression we Sanger sequenced an RT-PCR product using primers located in the skipped region of exon 3 and in exon 4 (primer sequences are available upon request) from the carrier (Fig. 2e). The data showed a monoallelic contribution of the wt allele, supporting that only the 3p(− 70) transcript is produced from the variant allele.

## Discussion and conclusions

We report two Danish families with a segregating *APC* missense variant. The variant is absent from all allele frequency databases, but was recently reported in a Chinese patient reported to have mild FAP, however no functional analyses were performed. Here we show that the variant creates a cryptic splice site resulting in a frameshift. We have data from four generations in both families, and although the families appear to be unrelated, they could have a common ancestor; however, since the variant has been detected in another patient from China, with a similar phenotype, it is unlikely to represent a Danish founder variant not related to AFAP. Although most published pathogenic *APC* mutations are truncating, disease-causing missense variants have been reported, but they are typically located in the exon-intron boundaries and result in splicing defects. The identified *APC* c.289G>A variant is located one third into exon 3 and creates a cryptic splice site, and several findings suggest that this variant is indeed pathogenic. Firstly, the phenotype and inheritance patterns in the families are consistent with *APC*-associated polyposis. Secondly, the variant segregates with disease in both families. Thirdly, our functional data confirm that the *APC* c.289G>A variant introduces a cryptic splice acceptor site that results in a major transcript isoform lacking 70 bp of exon 3, which is predicted to encode a truncated *APC* protein. Based on both the functional and clinical data we therefore classify the *APC* c.289G>A, p.(Gly97Arg) variant as pathogenic (class 5) in accordance with the ACMG-AMP guidelines for interpretation of sequence variants [10].

In family 1, individual II-III was – in addition to four colorectal cancers – also diagnosed with CD and an intrahepatic mucinous cystadenocarcinoma. CD is a rare congenital condition with cystic dilatations of the intrahepatic bile ducts. The condition is progressive, and the individuals typically develop symptoms later in life. CD can, among other symptoms, be characterized by recurrent cholangitis, cholelithiasis and gallbladder stones and is associated with an increased risk of cholangiocarcinoma, which affects up to 14% of the individuals. Different inheritance patterns and genes have been suggested to be involved in CD, i.e. autosomal recessive inheritance due to *PKHD1* mutations [11, 12]. In family 1 several family members suffered from cholelithiasis; the family might also have an autosomal dominant genetic predisposition to gall bladder disease, but we have no reason to believe that CD and the *APC* variant should be linked in any way, which is substantiated by the fact that the *APC* variant and gall bladder disease did not segregate in the family. On the other hand, it cannot be excluded that the *APC* variant could have contributed to the carcinogenesis of the CD-associated intrahepatic mucinous cystadenocarcinoma, however due to lack of tumour tissue, it has not been possible to perform *APC* loss of heterozygosity (LOH) analysis in the tumour.

The variant carriers in this study all have phenotypes consistent with AFAP; most had polyp count < 100 and onset of cancer at age 46–88, and although individual II-II in family 1 and individual III-I in family 2 both had large numbers of adenomas, they both developed cancer at a relatively high age (62-years-old and 78-years-old, respectively). This is in accordance with the association of a less severe phenotype and pathogenic variants in *APC* codons 1–177 [5–7]. On the other hand, an increasing amount of evidence suggests that modifier variants in other genes, low risk single nucleotide polymorphisms (SNPs) and non-genetic factors such as lifestyle influence the cancer risk and possibly also account for some of the intrafamilial variation [13]. Further research will help to elucidate the possible clinical implications of both the genotype and the role of modifying variants.

## Supplementary information

**Additional file 1: Supplementary Table S1.** Genes included in our gene panels. A) The research CRC gene panel. B) The clinical CRC gene panel.

## Data Availability

The data obtained during the current study are available from the corresponding author on reasonable request.

## Abbreviations

*APC*: Adenomatous Polyposis Coli
AFAP: Attenuated Familial Adenomatous Polyposis
FAP: Familial Adenomatous Polyposis
GAPPS: Gastric Adenocarcinoma and Proximal Polyposis of the Stomach
CRC: Colorectal Cancer
DNA: Deoxy Ribonucleic Acid
MMR: Mismatch Repair
*MLH1*: MutL homolog 1
*MSH2*: MutS homolog 2
*MSH6*: MutS homolog 6
*PMS2*: PostMeiotic Segregation increased 2
Gly: Glycine
Arg: Arginine
mRNA: Messenger Ribonucleic Acid
RNA: Ribonucleic Acid
RT-PCR: Reverse Transcription Polymerase Chain Reaction
CE: Capillary Electrophoresis
Bp: Base pairs
ACMG-AMP: American College of Medical Genetics and Genomics and the Association for Molecular Pathology
CD: Caroli Disease
*PKHD1*: Polycystic Kidney and Hepatic Disease 1
SNP: Single Nucleotide Polymorphism
AC: Adenocarcinoma
MCAC: Mucinous cystadenocarcinoma
IDC: Invasive ductal carcinoma
EEA: Endometrioid adenocarcinoma
n/a: Not available
Adn: Colonic adenomas
Duo: Duodenal cancer
LC: Hepatic cancer
MC: Breast cancer
EC: Endometrial cancer
PC: Pancreatic cancer
GC: Gastric cancer
Wt: Wild type
