# Supplementary material for: A rare missense variant in APC interrupts splicing and causes AFAP in two Danish families
Source: Hered Cancer Clin Pract. 2020 Apr 7;18:8. doi: 10.1186/s13053-020-00140-3 (PMC7140378; doi:10.1186/s13053-020-00140-3)
Supplement: Supplementary file 1 — Additional file 1: Supplementary Table S1. Genes included in our gene panels. A) The research CRC gene panel. B) The clinical CRC gene panel. [file 13053_2020_140_MOESM1_ESM.docx]

| **A) The research CRC gene panel** | |  | **B) The clinical CRC gene panel** | |
| --- | --- | --- | --- | --- |
| **Gene** | **NM nr.** |  | **Gene** | **NM nr.** |
| *APC* | NM_000038 |  | *APC* | NM_000038 |
| *AXIN2* | NM_004655 |  | *AXIN* | NM_004655 |
| *BLM* | NM_000057 |  | *BMPR1A* | NM_004329 |
| *BMPR1A* | NM_004329 |  | *EPCAM* | NM_002354 |
| *BRCA1* | NM_007294 |  | *GREM1* | NM_013372 |
| *BRCA2* | NM_000059 |  | *MLH1* | NM_000249 |
| *BUB1* | NM_004336 |  | *MSH2* | NM_000251 |
| *CDH1* | NM_004360 |  | *MSH3* | NM_002439 |
| *CHEK2* | NM_007194 |  | *MSH6* | NM_000179 |
| *EPCAM* | NM_002354 |  | *MUTYH* | NM_012222 |
| *EXO1* | NM_006027 |  | *NTHL1* | NM_002528 |
| *FAN1* | NM_014967 |  | *PMS2* | NM_000535 |
| *FOCAD* | NM_017794 |  | *POLD1* | NM_002691 |
| *GALNT12* | NM_024642 |  | *POLE* | NM_006231 |
| *GREM1* | NM_013372 |  | *PTEN* | NM_000314 |
| *IPMK* | NM_152230 |  | *SMAD4* | NM_005359 |
| *MLH1* | NM_000249 |  | *STK11* | NM_000455 |
| *MLH3* | NM_014381 |  |  |  |
| *MSH2* | NM_000251 |  |  |  |
| *MSH3* | NM_002439 |  |  |  |
| *MSH6* | NM_000179 |  |  |  |
| *MUTYH* | NM_012222 |  |  |  |
| *NTHL1* | NM_002528 |  |  |  |
| *PMS1* | NM_000534 |  |  |  |
| *PMS2* | NM_000535 |  |  |  |
| *POLD1* | NM_002691 |  |  |  |
| *POLE* | NM_006231 |  |  |  |
| *PTEN* | NM_000314 |  |  |  |
| *RINT1* | NM_021930 |  |  |  |
| *RPS20* | NM_001146227 |  |  |  |
| *SMAD4* | NM_005359 |  |  |  |
| *SMAD9* | NM_001127217 |  |  |  |
| *STK11* | NM_000455 |  |  |  |
| *TP53* | NM_000546 |  |  |  |
